# What hematological and endocrinal indicators are important in COVID-19 infection?

**DOI:** 10.34172/hpp.2022.26

**Published:** 2022-08-20

**Authors:** Alireza Ostadrahimi, Vahideh Sadra, Amir Bahrami, Zohreh Razzaghi, Mostafa Najafipour, Helda Tutunchi, Farzad Najafipour

**Affiliations:** ^1^Nutrition Research Center, Department of Clinical Nutrition, School of Nutrition and Food Sciences, Tabriz University of Medical Sciences, Tabriz, Iran; ^2^Endocrine Research Center, Tabriz University of Medical Sciences, Tabriz, Iran; ^3^Department of Internal Medicine, Faculty of Medicine, Ardabil Azad University of Medical Sciences, Ardabil, Iran

**Keywords:** Coronavirus, Endocrine system, Hematology, Triiodothyronine, Free vitamin D

## Abstract

**Background:** Clinical evidence of endocrine involvement in coronavirus disease needs further investigation. The aim of the present study was to assess the relationship between hematology and endocrine parameters in coronavirus disease 2019 (COVID-19) infection.

**Methods:** In the present cross-sectional study, a total of 320 patients (215 survivors and 105 non-survivors) with confirmed COVID-19 infection were enrolled. After isolation of serum samples, hematological, biochemical, and hormonal parameters were analyzed.

**Results:** The mean age of survivors and non-survivors was 58.92 (SD: 15.28) and 63.65 (SD: 16.62) years, respectively. The results demonstrated significant differences in free triiodothyronine (FT3) [MD (95% CI): 0.40 (0.10, 0.71), *P*=0.009], total calcium [MD (95% CI): 0.53 (0.21, 0.86), *P*=0.003], vitamin D [MD (95% CI): 7.72 (6.38, 9.05), *P*=0.003], erythrocyte sedimentation rate (ESR) [MD (95% CI): 17.09 (9.38, 22.05), *P*=0.004) and serum ferritin [Median difference: -1091.9, *P*<0.001), between survivors and non-survivors, respectively.

**Conclusion:** The results revealed that some hematological and endocrine factors play an important role in prognosis of COVID-19 infection. However, further studies with a larger population are required to clarify the exact effects of COVID-19 on the endocrine system.

## Introduction

 Coronavirus disease 2019 (COVID-19) is a new infectious disease that was first reported in Wuhan China in 2019, and rapidly spread all around the world. The recent virus belongs to a large group of ribonucleic acid (RNA) viruses known as the coronavirus family.^1^ The broad manifestation of the disease includes the common cold to more severe conditions like pneumonia, respiratory, multi-organ failure, and death.^1,2^ However, endocrinopathy of disease has not yet been detected.^2^ The pathogenesis of the COVID-19 disease is based on severe acute respiratory syndrome coronavirus 2 (SARS-CoV-2) entry via angiotensin-converting enzyme 2** (**ACE2) receptors in pneumocytes and other cells.^3^ Numerous endocrine organs such as pancreas, thyroid, testis, ovary, adrenal, and pituitary gland express ACE2 receptors.^4^

 An important question that the American Thyroid Association (ATA) has not yet replied, is concerned about how the newfound coronavirus pandemic might influence people with thyroid disorders.^5^ Most of the information about coronavirus and thyroid gland belongs to the results of observational studies of the SARS outbreak in 2003.^4-6^ Bacterial and viral infections are more common and difficult to control in diabetes patients. Numerous reports indicate that COVID-19 patients with diabetes have a higher morbidity and mortality rate than non-diabetic patients. Several mechanisms have been suggested to increase mortality in these patients such as increased insulin resistance in type 2 diabetes mellitus (T2DM) which is induced by a cytokine storm.^7^ Moreover, obesity which is more common in T2DM patients could be related to severe disease in COVID-19. Additionally, low levels of serum T3 and T4 have been reported in patients with SARS, so that the more severe disease the lower the T3 level, implying an underlying sick euthyroid syndrome.^8^

 Vitamin D status affects the immune system which has been documented in many observational studies. Vitamin D increases cellular immunity by decreasing the cytokine storm which is induced by the innate immune system. A study has revealed that 25-OH vitamin D level above 50 ng/ml was associated with a 27% reduction in influenza-like illnesses, versus vitamin D level lower than 20 ng/mL.^9,10^ A growing body of evidence suggests that COVID-19 can cause hematological changes in infected patients. Anemia, lymphopenia, thrombocytopenia, leukocytosis, and neutrophilia are commonly observed hematological abnormalities in COVID-19, which are more common in severe patients requiring hospitalization. Therefore, hematological parameters may serve as a prognostic indicator in patients with COVID-19.^11,12^

 Although some previous studies have investigated the association of COVID-19 infection with endocrine system,^5,6^ overall clinical evidence of endocrine involvement in patients with COVID-19 needs further consideration. Therefore, in the present cross-sectional study, we aimed to investigate the relationship between hematology and endocrine parameters in COVID-19 infection.

## Materials and Methods

###  Participants

 In the present cross-sectional study, 320 patients (215 survivors and 105 non-survivors) with laboratory-confirmed SARS-CoV-2 who were admitted to Imam Reza hospital of Tabriz University of Medical Sciences for 6 months were recruited. COVID-19 infection was confirmed based on positive real-time polymerase chain reaction (RT-PCR) of nasopharyngeal swab and/or imaging findings consistent with COVID-19 pneumonia on chest computed tomography (CT) scan and ruling out other causes of pneumonia. Exclusion criteria were history of adrenal, thyroid, and pituitary disorders and glucocorticoids and thyroid drug consumption. The severity of COVID-19 was assessed based on the mortality of the disease.

###  Sample size

 Assuming 18% mortality rate for non-diabetic COVID-19 patients, and 40% for diabetic COVID-19 patients based on the previous study^13^ and assuming a sample size ratio of 1.5 for non-diabetic to the diabetic group, the study would require a sample size of 215 for survivors and 105 for non-survivors group to achieve a power of 90% for detecting a difference in mortality rate of 0.22 between the two groups at a two sided *P* value of 0.05.

###  Assessments

 Whole blood was obtained from patients at the time of admission in fasting state and serum samples were separated from whole blood and stored at -70^o^C until analysis. Thyroid-stimulating hormone (TSH), FT3, free thyroxine (FT4), and cortisol were measured by an electronic chemiluminescence immunoassay (Elecsys Roche Diagnostics, Mannheim, Germany). Calcium was measured with an ion-selective electrode (9180 model; Basel, Switzerland). Ferritin was measured by a two-site immunoluminometric assay (Byk-Sangtec Diagnostica; Dietzenbach, Germany). Blood glucose was determined by photometry assay (Pars Azmun, Iran). Serum 25-hydroxyvitamin D [25(OH) D] levels were assessed by chemiluminescence’s immunoassay on the automated LIAISON^®^ analyzer (Stillwater, MN). Erythrocyte sedimentation rate (ESR) was measured using the conventional Westergren method. Weight and height were measured according to the standard procedures and body mass index (BMI) was calculated as weight (kg)/height (m^2^).

###  Statistical analysis

 Statistical analysis was performed using IBM SPSS Statistics software (IBM SPSS Statistics, Armonk, USA, version 23). Kolmogorov-Smirnov test was used to check the normality of the data distribution. Data were expressed as mean (SD) for continuous variables and frequency (percentage) for categorical variables, and median (25^th^, 75^th^) for values with skewed distribution. An independent sample *t* test was used for comparing means between two groups. Data with non-normal distribution were analyzed using the Mann-Whitney U test. All statistical tests were two-sided, and *P* values < 0.05 were considered statistically significant.

## Results

 In the present cross-sectional study, a total of 320 patients (215 survivors and 105 non-survivors) with COVID-19 referred to Imam Reza hospital at Tabriz University of Medical Sciences were enrolled. Despite the different sample sizes of the studied group, 56.27% and 58.09% of survivors and non-survivors were men, respectively. The average age of survivors and non-survivor patients was 58.92 (SD: 15.28) and 63.65 (SD: 16.62) years (*P* = 0.070), respectively. Moreover, the frequency of type 2 diabetic patients in survivor and non-survivors were 38 (17.66%) and 28 (26.65%), respectively (*P* = 0.211). After adjusting for age, sex and BMI as confounding variables, the mortality rate in diabetic patients was 62% higher than non-diabetic counterparts (OR = 1.62, 95% CI: 0.8-3.26, *P* = 0.170).

 As shown in Table 1, a significantly elevated white blood cells (WBCs) count (11.97 ± 4.05 versus 9.13 ± 3.51 10^3^/µL, *P* = 0.016) and ESR (54.31 ± 23.98 versus 37.22 ± 16.55 mm, *P* = 0.004) was observed in non-survivors compared to survivors. In contrast, hemoglobin levels in survivors were significantly higher compared to non-survivors (12.99 ± 2.45 versus 11.67 ± 2.74 g/dL, *P* = 0.002). Furthermore, a significantly elevated BMI (27.78 ± 2.60 kg/m^2^ versus 26.59 ± 2.41 kg/m^2^, *P* = 0.005) as well as glycosylated hemoglobin (HbA1C) (5.84 ± 1.68% versus 5.32 ± 1.31%, *P* = 0.035) was observed in non-survivors compared to survivors.

**Table 1:** Comparison of basic and hematological parameters between survivor and non-survivor patients

	**Survivors** **(n=215)**	**Non-survivors** **(n=105)**	***P*** ** value**
Age (y)	58.92 (15.28)	63.65(16.62)	0.074
Gender (male), No. (%)	121 (56.27)	61 (58.09)	0.499
BMI (kg/m^2^)	26.59 (2.41)	27.78 (2.60)	**0.005**
Type 2 diabetes, No. (%)	38 (17.66)	28 (26.65)	0.211
WBC (10^3^/µL)	9.13(3.51)	11.97 (4.05)	**0.016**
Platelet (10^3^/µL)	210.40 (101.77)	194.40 (88.43)	0.183
Hb (g/dL)	12.99 (2.45)	11.67 (2.74)	**0.002**
ESR (mm)	37.22 (16.55)	54.31 (23.98)	**0.004**
HbA1C%	5.32 (1.31)	5.84 (1.68)	**0.035**

WBC: white blood cells; Hb: hemoglobin; ESR: erythrocyte sedimentation rate. Data are presented as mean (SD) or number (%). *P*<0.05 was considered as statistically significant.

Table 2 presents the biochemical parameters differences between survivors and non-survivor patients with COVID-19 infection. Blood urea [40 (25, 55) versus 68.5 (40.75, 107.75) mg/dL, *P*<0.001], and creatinine [1.10 (0.87, 1.32) versus 1.43 (1, 2.66) mg/dL, *P* = 0.002] levels were significantly higher in non-survivors. However, ferritin [207.8 (73.6, 772.4) versus 1299.7 (314.2, 1575) ng/mL, *P*<0.001] and total calcium (8.85 ± 0.99 versus 8.31 ± 0.83 mg/dL, *P* = 0.003) levels were significantly higher in mentioned group compared to non-survivors.

**Table 2:** Comparison of biochemical parameters between survivor and non-survivor patients

	**Survivors (n=215)**	**Non-survivors (n=100)**	**MD (95% CI) **	***P*** ** value**
BG (mg/dL)	136.26 (65.87)	150.52 (73.56)	-14.26 (-30.48, 1.96)	0.083^a^
Urea (mg/dL)	40 (25, 55)	68.50 (40.75, 107.75)	-28.50	**<0.001** ^b^
Creatinine (mg/dL)	1.10 (0.87, 1.32)	1.43 (1, 2.66)	-0.33	**0.001** ^b^
Ferritin (ng/ml)	207.81 (73.61, 772.4)	1299.70 (314.20, 1575)	-1091.9	**<0.0001** ^b^
Positive CRP (mg/dL)	1.69 (0.82)	1.51(0.70)	0.18 (-0.006,0.36)	0.059^a^
Total calcium (mg/dL)	8.85(0.99)	8.31(0.83)	0.53 (0.21,0.86)	**0.003** ^a^

BG: blood glucose; hs-CRP: high-sensitive C-reactive protein. Mean (SD) and mean difference (95% CI) are presented for normally distributed data; Median (25th and 75th percentiles) and median difference are presented for data not normally distributed. ^a^ *P* value based on independent samples *t* test. ^b^ *P* value based on Mann-Whitney U test. *P*<0.05 was considered as statistically significant.

 Vitamin D and hormonal differences in survivors and non-survivors with SARS-COVID-19 infection are also presented in Table 3. Survivors showed significantly higher vitamin D levels compared to non-survivors (22.01 ± 6.79 versus 14.29 ± 7.02 ng/ml, *P* = 0.003). Additionally, free T_3_ (FT_3_) was also significantly lower in non-survivors (3.84 ± 0.91 versus 3.44 ± 0.89 ng/dL, *P* = 0.009). No significant differences were observed regarding the other hematological, biochemical, and hormonal parameters between the studied groups.

**Table 3:** Comparison of vitamin D and hormonal changes between survivor and non-survivor patients

	**Survivors (n=115)**	**Non-survivors (n=50)**	**MD (95% CI)**	***P*** ** value**
Vitamin D (ng/mL)	22.01 ± 6.79	14.29 ± 7.02	7.72 (6.38,9.05)	**0.003** ^a^
Cortisol (ng/dL)	473.38 ± 210.06	527.74 ± 176.88	-54.36 (-121.57,12.85)	0.112^a^
TSH (µIU/mL)	0.60 (0.2, 1.35)	0.35 (0.4, 1.4)	0.25	0.121^b^
FT_3_ (ng/dL)	3.84 ± 0.91	3.44 ± 0.89	0.40 (0.10,0.71)	**0.009** ^a^
FT_4_ (ng/dL)	2.16 ± 1.26	2.38 ± 1.01	-0.21 (-0.62,0.18)	0.283^a^

TSH: thyroid stimulating hormone; FT_3_: free T_3_; FT_4_: free T_4_ Mean (SD) and Mean difference (95% CI) are presented for normally distributed data; Median (25th and 75th percentiles) and Median difference are presented for data not normally distributed. ^a^ *P* value based on independent samples *t* test. ^b^ *P* value based on Mann-Whitney U test. *P*<0.05 was considered as statistically significant.

## Discussion

 In the present study, the association of COVID-19 infection with the endocrine system was explored. Moreover, its associations with hematological and biochemical parameters were also assessed as secondary endpoints. The results point out that serum vitamin D and FT3 levels were lower in non-survivors compared with survivors of COVID-19. However, Serum levels of TSH, FT4, and cortisol did not predict mortality. To the best of our knowledge, limited data is available about cortisol and thyroid hormones level in patients with COVID-19. In the study of Leow et al in 2005,^14^ the 39.3% of patients had evidence of hypocortisolism in 3 months after recovery. Although no radionuclide scans or thyroid biopsies were performed, the authors postulated that SARS induced thyroiditis or hypophysitis, either alone or in combination, was the most probable reason for the thyroid and hypothalamic-pituitary-adrenal dysfunction. A recent cohort study^15^ revealed that a doubling of cortisol concentration was associated with a significant increase in the hazard of mortality, and a decreased median survival, which is the marker of the severity of illness.^16^ In that study, cortisol was a better independent predictor than other laboratory markers related to COVID-19 infection.

 An autopsy study in five patients with SARS has shown marked destruction of the follicular and parafollicular thyroid cells.^7^ A retrospective study on 50 patients with COVID-19 demonstrated that the serum TSH and total T3 levels were significantly lower in COVID-19 patients compared with a healthy control group and non-COVID-19 pneumonia patients. The more severe disease, the lower TSH and T3 levels were, with statistical significance.^17^ There might be a unique effect of COVID-19 on TSH-secreting cells. Two possible mechanisms were suggested for these changes. One is a direct effect of the virus on the pituitary cells, another is an indirect effect of various systemic changes mediated by induction of different pro-inflammatory cytokines caused by the viral infection.^17,18^

 In this study, we showed that high blood sugar in patients with COVID-19 infection is associated with a higher risk of death compared to patients with normal blood sugar. The link between diabetes and infection and increased mortality has been known in the past. Several mechanisms have been suggested for worsening blood sugar in COVID-19 infection. ACE2 receptor is expressed in the pancreas more than in the lungs. SARS-CoV mediated acute diabetes due to pancreatic β cell damage has previously been reported. It seems that SARS-CoV and perhaps SARS-CoV2 could be considered as potential environmental triggers for the development of type 1 diabetes mellitus.^19,20^ Hypokalemia in COVID-19 can also worsen glucose control in patients with preexisting diabetes. Hyperglycemia could be associated with severe disease and increased mortality. Higher levels of serum inflammatory biomarkers such as interleukin-6 (IL-6), and C-reactive protein (CRP), serum ferritin, and coagulation factors (D-Dimer) have also been reported in patients with diabetes mellitus and COVID-19, concluding that people with diabetes are more susceptible to cytokine storm leading to acute respiratory distress syndrome (ARDS).^21^

 In our study, BMI was higher in non-survivors compared to survivors. Obesity leads to worse blood sugar and increased mortality in COVID-19 patients. Obese individuals have higher adipose tissue with higher ACE2 expression. Additionally, several pro-inflammatory biomarkers such as tumor necrosis factor-α (TNF-α), IL-6, and monocyte chemoattractant protein-1 (MCP-1) produced by visceral and subcutaneous fat are higher in obese diabetic patients.^22,23^

 In agreement with our findings, a multicenter study in 20 European countries reported that serum vitamin D levels were significantly lower in expired patient’s than alive patients, especially in Spain, Italy, and Switzerland.^10^ Vitamin D deficiency is associated with increased autoimmunity and infection susceptibility. Vitamin D increases cellular immunity by reducing the cytokine storm induced by the innate immune system. Furthermore, vitamin D reduces the risk of respiratory tract infection through three mechanisms including, (*i*) maintaining tight junctions, (*ii*) killing enveloped viruses through cathelicidin, IL-37, and induction of defenses, and (*iii*) reducing the production of pro-inflammatory cytokines.^9,24^

 The result of the present study also reports severe anemia and elevated ferritin levels in non-survivors compared to survivors. In line with our finding, a retrospective cohort study on 191 patients with COVID-19 in China demonstrated that non-survivors had a significantly higher serum ferritin level compared to survivors.^25^ A systematic review and meta-analysis evaluating biomarkers of anemia and iron metabolism in patients with COVID-19 showed that hemoglobin levels were lower with older age, overall comorbidities, and critically ill patients.^26^ It has been proposed that viral proteins induce hemolysis and form a complex with the released heme, resulting in dysfunctional hemoglobin, with decreased oxygen and CO_2_ transport, producing symptoms of respiratory distress.^11^

 Compared with survivors, non-survivors had a significantly higher WBC count in the current study. Laboratory findings on COVID-19 patients revealed that the vast majority of patients presented with lymphopenia (83.2%); whereas, 36.2% and 33.7% had thrombocytopenia and leukopenia, respectively. These hematological abnormalities were more prominent among severe versus none severe cases.^12^ Wang et al^27^ also highlighted an association between lymphopenia and the need for intensive care unit care. Additionally, Zhao et al^28^ showed an association between lymphopenia and ARDS development. Moreover, the results of four descriptive studies in china showed an increased risk of ARDS which was significantly associated with decreased lymphocyte count and increased neutrophils.^7,27^ A meta-analysis of 10 studies revealed that severe disease was associated with lymphopenia and higher leukocyte counts.^29^ In addition, the study of Yamada et al^30^ showed that leukocytosis was prominent in severe cases.

 A meta-analysis on 1779 COVID-19 patients reported significantly lower platelet counts in severe cases.^31^ Although non-survivors had lower platelet counts than survivors in our study, this difference was not statistically significant. Differences between studies might be related to the time of the tests. Moreover, treatment with drugs that can cause thrombocytopenia was started in most countries, possibly contributing to the discrepancies between the results.^32^

 In our study, ESR, creatinine, and CRP levels were significantly higher in non-survivors compared to survivors. The findings of a pooled analysis suggest that in severe COVID-19 cases elevated ESR levels are more prominent than none severe cases reflecting the more profound inflammatory response.^33^

 The prevalence of direct kidney involvement in COVID-19 patients is low but it is known as a marker of severe disease and predicts mortality.^34^ A systemic review and meta-analysis of 24 studies involving 4963 COVID-19 patients revealed acute kidney injury (AKI) was associated with severity and mortality. The incidence of AKI was (52.9% vs 0.7%) in none survivors and survivors and continuous renal replacement therapy was needed in 5.6% of severe cases and 0.1% of none severe cases.^35^

 Total calcium level was also lower in non-survivors compared to survivors in the present study. Hypocalcemia was detected in 70% of patients hospitalized with SARS infection in 2003.^36^ Recent studies on COVID-19 and calcium homeostasis revealed that hospitalized COVID-19 patients had significantly lower ionized calcium levels compared to none hospitalized, and lower serum calcium levels were associated with clinical severity, poor prognosis, and a higher rate of mortality.^37^

 The study limitations are as follows: using only one therapeutic center for recruiting patients and due to the cross-sectional design of this study, the causality effect could not be indicated. However, these results are shared to inform the long-term functional of COVID-19 patients who needed hospital admission.

## Conclusion

 The results revealed that some hematological and endocrine factors play an important role in prognosis of COVID-19 infection. However, further studies with a larger population are required to clarify the association of COVID-19 infection with the endocrine system and mortality.

## Acknowledgments

 We sincerely thank the patients who participated in the present study.

## Authors’ contributions

 AO, VS, AB, and FN designed research and contributed to the conception of the project, development of overall research plan, and study oversight. HT, VS, ZR, and MN drafted the manuscript and analyzed and interpreted the data. FN and HT contributed to the final revision of the manuscript. All authors approved the final version of this manuscript.

## Funding

 The study was financially supported by the Endocrine Research Center of Tabriz University of Medical Sciences.

## Data availability statement

 The data that support the findings of this study are available on request from the corresponding author.

## Ethical approval

 The study was approved by the ethics committee of Tabriz University of Medical Sciences (Code: IR.TBZMED.REC.1399.100). Written informed consent was obtained from all individuals before the study.

## Competing interests

 All authors declare that there is no conflict of interest.

## Disclaimer

 The authors claim that no part of this paper is copied from other sources.
